# Incidence and impact of extra-pulmonary organ failures on hospital mortality in acute exacerbation of idiopathic pulmonary fibrosis

**DOI:** 10.1038/s41598-020-67598-y

**Published:** 2020-07-01

**Authors:** Yi Zhuang, Ying Zhou, Xiaohua Qiu, Yonglong Xiao, Hourong Cai, Jinghong Dai

**Affiliations:** Department of Pulmonary and Critical Care Medicine, Nanjing Drum Tower Hospital, Clinical College of Nanjing Medical School, No. 321 Zhongshan Road, Nanjing, 210008 Jiangsu China

**Keywords:** Diseases, Risk factors

## Abstract

To evaluate hospital mortality and associated risk factors for acute exacerbations of idiopathic pulmonary fibrosis (AEIPF). Emphases were put on incidence and impact of extra-pulmonary organ failures. Patients diagnosed with AEIPF from July 2014 to September 2018 were enrolled. Clinical data were collected. Acute physiology and chronic health evaluation II (APACHE II) and simplified acute physiological score II (SAPS II) were calculated. Extra-pulmonary organ failures were diagnosed upon criteria of sequential organ failure assessment (SOFA). Forty-five patients with AEIPF were included. Eighteen patients (40.0%) developed extra-pulmonary organ failures, and 25 patients (55.6%) died during hospitalization. Serum C-reactive protein (CRP) (*p* = 0.001), SAPS II (*p* = 0.004), SOFA (*p* = 0.001) were higher, whereas arterial oxygen pressure (PaO_2_)/ fractional inspired oxygen (FiO_2_) (*p* = 0.001) was lower in non-survivors than survivors. More non-survivors developed extra-pulmonary organ failures than survivors (*p* = 0.002). After adjustment, elevated serum CRP (OR 1.038, *p* = 0.049) and extra-pulmonary organ failure (OR 13.126, *p* = 0.016) were independent predictors of hospital mortality in AEIPF. AEIPF had high hospital mortality and occurrence of extra-pulmonary organ failure was common. Elevated serum CRP and extra-pulmonary organ failure had predictive values for mortality.

## Introduction

Idiopathic pulmonary fibrosis (IPF) is a chronic, progressive and fatal fibrosing interstitial pneumonia with unknown etiology^1^. The clinical course of IPF is highly heterogeneous and unpredictable. For some patients, the disease remains stable or progresses slowly over years. But a small amount of patients may develop sudden exacerbations of respiratory function impairment, referred to as acute exacerbations of idiopathic pulmonary fibrosis (AEIPF)^2^, resulting in refractory hypoxemia and respiratory failure. AEIPF is the leading cause of death in IPF, with the hospital mortality up to 60%^2,3^.

AEIPF shared similar pathophysiological characteristics and clinical need with another severe condition, acute respiratory distress syndrome (ARDS)^4^. A majority of patients with AEIPF needed admission to intensive care unit (ICU) for severe complications and multiple organ failures. AEIPF was also considered as an inflammation associated disease^5^. Despite the high hospital mortality rates, the prognostic factors for short-term mortality in patients with AEIPF remained uncertain. Few studies described the conditions of extra-pulmonary organs in patients with AEIPF. Therefore, we conducted this retrospective study in an interstitial lung disease center in China, to investigate the hospital mortality and associated risk factors of hospital mortality in patients with AEIPF. Acute physiology and chronic health evaluation II (APACHE II)^6^, simplified acute physiological score II (SAPS II)^7^ and sequential organ failure assessment (SOFA) system^8^ were used to evaluate the organ conditions and assess the incidence and impact of extra-pulmonary organ failures on hospital mortality in AEIPF.

## Results

### Patients inclusion

**Figure 1:**
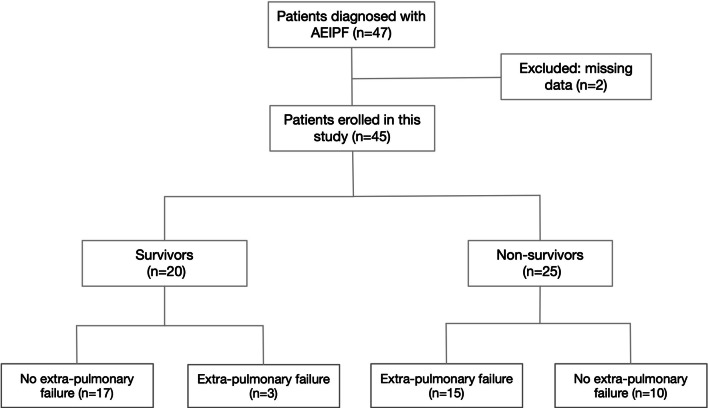
Flowchart of patients’ selection. AEIPF = acute exacerbation of idiopathic pulmonary fibrosis.

There were 47 consecutive patients diagnosed with AEIPF in our center during the study period. Two patients missing follow-up data were excluded. Hence, a total of 45 patients were enrolled (Fig. 1). They were 36 males and 9 females with a mean age of 66.6 ± 9.0 years old (range 42–82 years old). Twenty-two (48.9%) patients were smokers. They developed AE in 21.9 ± 17.6 months (range 0–84 months) from the diagnosis of IPF. Before the occurrence of AEIPF, 14 patients (31.1%) had received corticosteroids treatment for 11.9 ± 11.3 months (range 3–36 months) and 2 patients (4.4%) were on pirfenidone or nintedanib therapy for 5.5 ± 2.5 months (range, 3-8 months).

**Figure 2:**
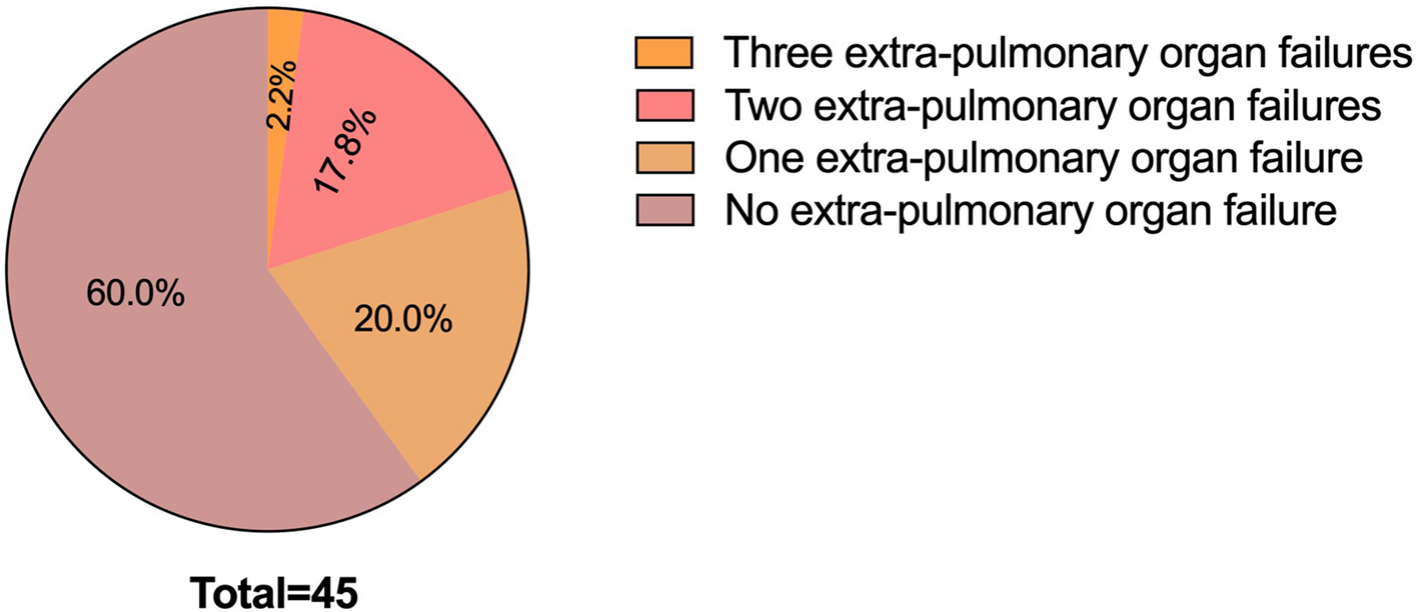
A breakdown of presence of extra-pulmonary organ failure in AEIPF patients. Of the 45 patients, one patient (1/45, 2.2%) combined with three extra-pulmonary organ failures, eight patients (8/45, 17.8%) had two extra-pulmonary organ failures, nine patients (9/45, 20.0%) had one extra-pulmonary organ failure, and 27 patients (27/45, 60.0%) combined with no extra-pulmonary organ failure. AEIPF = acute exacerbation of idiopathic pulmonary fibrosis.

At admission, the mean arterial oxygen pressure (PaO_2_)/ fractional inspired oxygen (FiO_2_) was 158.0 ± 60.3 mmHg (range 45.0–282.8 mmHg). Extra-pulmonary organ failures were observed in 18 patients (40.0%). Among them, 13 patients (13/18, 72.2%) had acute cardiovascular failure, and 5 of them were treated with vasopressors infusion. Six patients (6/18, 33.3%) had acute liver failure and 4 patients (4/18, 22.2%) had acute kidney failure. One patient (1/18, 5.6%) had 3 extra-pulmonary organ failures, 8 patients (8/18, 44.4%) had 2 extra-pulmonary organ failures, and 9 patients (9/18, 50.0%) had 1 extra-pulmonary organ failure (Fig. 2). High-dose corticosteroids (0.5–1 g/d methylprednisolone or its equivalent) for 3–5 days was used in 26 patients (57.8%) after diagnosis. Non-invasive ventilation (NIV) was used in 25 patients (55.6%), and 2 of them were performed invasive mechanical ventilation (IMV). Twenty-five patients died in the hospital with a mortality rate of 55.6% (25/45) and 20 patients discharged. The mean length of hospital stay was 14.7 ± 8.8 days (range 3–35 days). Thirty-four patients (75.6%) had stayed in respiratory intensive care unit (RICU) for 10.1 ± 9.9 days (range 2–35 days).

## Comparison of clinical characteristics between survivors and non-survivors

As presented in Table 1, serum C-reactive protein (CRP) level (66.8 ± 53.6 mg/L vs. 19.6 ± 18.4 mg/L, *p* = 0.001), SAPS II score (31.8 ± 6.2 vs. 26.8 ± 4.9, *p* = 0.004) and SOFA score (3.9 ± 1.2 vs. 2.8 ± 0.7, *p* = 0.001) were higher in non-survivors than those in survivors, whereas PaO_2_/FiO_2_ (131.7 ± 51.3 vs. 190.9 ± 55.3, *p* = 0.001) was lower in non-survivors than that in survivors. Non-survivors were more likely to develop extra-pulmonary organ failures than survivors (60.0% vs. 15.0%, *p* = 0.002). More non-survivors received NIV than survivors (88.0% vs. 15.0%, *p* < 0.001). No significant differences were observed in age, gender, smoking status, chest high-resolution computerized tomography (HRCT) pattern, APACHE II score or high-dose steroids usage between the two groups.

**Table 1:** Comparison of clinical characteristics between non-survivors and survivors in patients with AEIPF.

Variables	Non-survivors (n = 25)	Survivors (n = 20)	*p*
Age, years	68.2 ± 9.0	64.7 ± 8.8	0.197
Male, n (%)	20 (80.0)	16 (80.0)	> 0.999
Smoker, n (%)	13 (52.0)	9 (45.0)	0.641
Pre-admission steroid usage, n (%)	6 (24.0)	8 (40.0)	0.249
Pirfenidone/ nintedanib usage, n (%)	2 (8.0)	0 (0.0)	0.495
Duration from diagnosis of IPF to AE, months	20.0 ± 19.5	24.2 ± 15.1	0.432
Fever, n (%)	9 (36.0)	7 (35.0)	0.944
PaO_2_/FiO_2_, mmHg	131.7 ± 51.3	190.9 ± 55.3	0.001
WBC, *10^9^/L	12.0 ± 5.7	9.7 ± 2.7	0.101
CRP, mg/L	66.8 ± 53.6	19.6 ± 18.4	0.001
LDH, U/L	506.8 ± 204.7	469.7 ± 146.1	0.108
**HRCT pattern, n**			
Peripheral/multifocal/diffuse	4/6/15	3/4/13	0.936
Presence of extra-pulmonary organ failure, n (%)	15(60.0)	3 (15.0)	0.002
APACHE II score	17.6 ± 3.9	15.3 ± 4.5	0.075
SAPS II score	31.8 ± 6.2	26.8 ± 4.9	0.004
SOFA score	3.9 ± 1.2	2.8 ± 0.7	0.001
**Treatment, n (%)**			
High-dose steroids	17 (68.0)	9 (45.0)	0.121
NIV	22 (88.0)	3 (15.0)	< 0.001
IMV	2 (8.0)	0 (0.0)	0.495
Lengths of stay in RICU, days	9.2 ± 8.4	8.7 ± 10.5	0.135
Lengths of stay in hospital, days	11.7 ± 8.9	19.5 ± 7.0	0.001

AE-IPF = acute exacerbation of idiopathic pulmonary fibrosis; PaO_2_ = arterial oxygen pressure; FiO_2_ = fractional inspired oxygen; WBC = white blood cell; CRP = C-reactive protein; HRCT = high-resolution computed tomography; APACHE II = Acute physiology and chronic health evaluation II; SAPS II = simplified acute physiological score II; SOFA II = sequential organ failure assessment; NIV = non-invasive mechanical ventilation; IMV = Invasive mechanical ventilation; RICU = respiratory intensive care unit.

## Risk factors on hospital mortality

**Figure 3:**
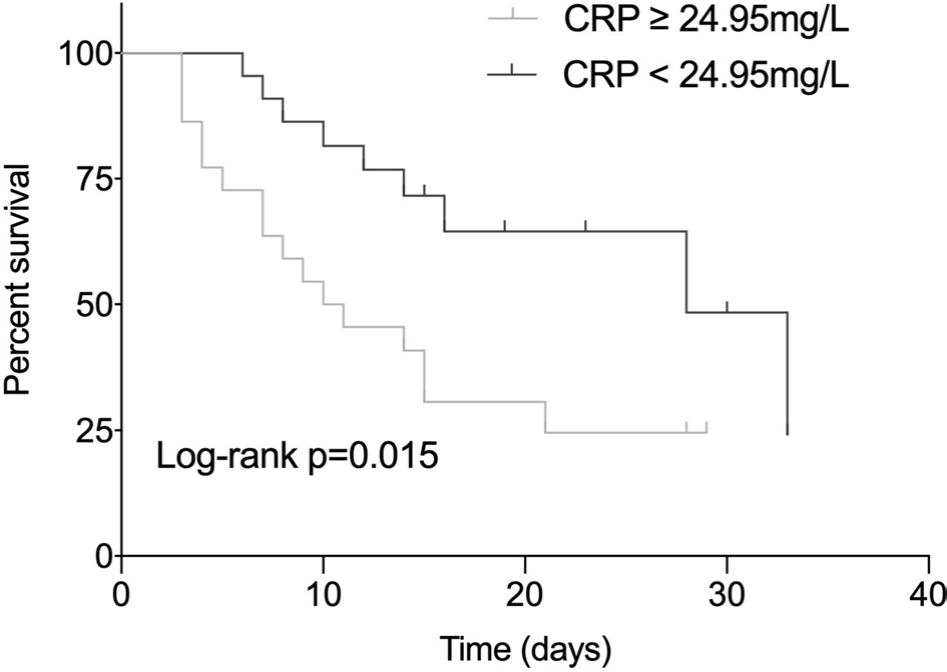
Kaplan–Meier survival estimate of AEIPF patients stratified according to serum CRP levels. AEIPF = acute exacerbation of idiopathic pulmonary fibrosis; CRP = C-reactive protein.

**Figure 4:**
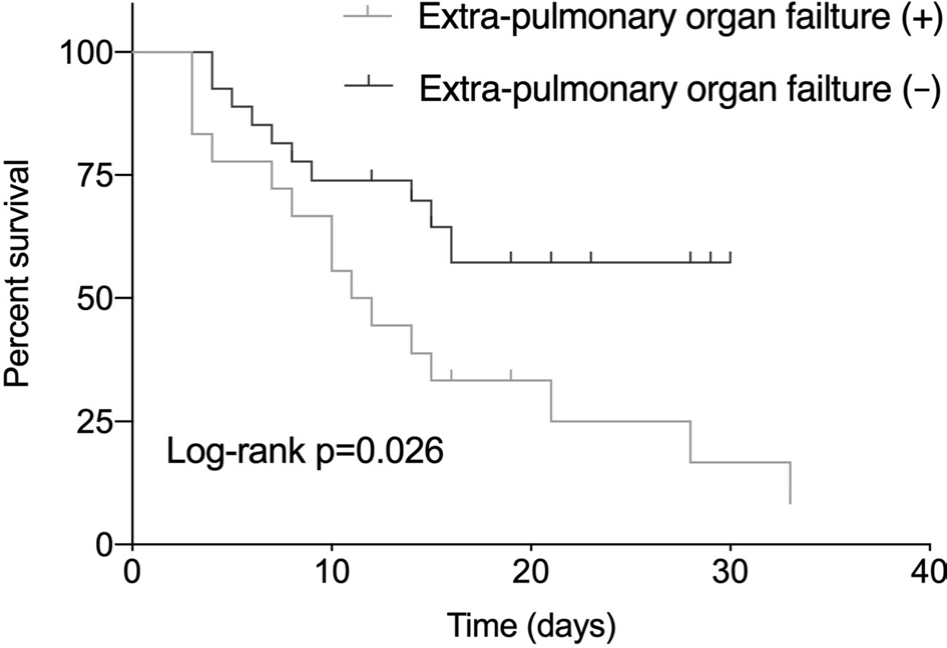
Kaplan–Meier survival estimate of AEIPF patients stratified according to extra-pulmonary organ failure. AEIPF = acute exacerbation of idiopathic pulmonary fibrosis.

In univariate analysis, PaO_2_/FiO_2_ (OR 0.980; 95% CI 0.967–0.993; *p* = 0.014), serum CRP (OR 1.040; 95% CI 1.011–1.070; *p* = 0.006), presence of extra-pulmonary organ failure (OR 8.500; 95% CI 1.964–36.790; *p* = 0.004), SAPS II score (OR 1.184; 95% CI 1.039–1.349; *p* = 0.011) and SOFA score (OR 3.585; 95% CI 1.522–8.443; *p* = 0.003) were associated with hospital mortality. After adjustment for age and gender, multivariate analysis revealed that an elevated serum CRP (OR 1.038; 95% CI 1.001–1.076; *p* = 0.049) and extra-pulmonary organ failure (OR 13.126; 95% CI 1.608–107.161; *p* = 0.016) were independent risk factors for hospital mortality in patients with AEIPF (Table 2, Fig. 3 and Fig. 4).

**Table 2:** Logistic regression analysis of risk factors of hospital mortality in AEIPF patients.

	Univariate			Multivariate		
	OR	95% CI	*p*	OR	95% CI	*p*
Age	1.047	0.976–1.122	0.199	0.948	0.838–1.072	0.394
Sex	0.571	0.131–2.491	0.456	4.237	0.381–47.175	0.240
Even smoker	1.186	0.568–2.475	0.650			
Pre-admission steroids usage	0.898	0.342–2.358	0.826			
Duration from diagnosis of IPF to AE	0.986	0.953–1.021	0.426			
PaO_2_/FiO_2_	0.980	0.967–0.993	0.014	0.983	0.962–1.003	0.101
WBC	1.157	0.966–1.387	0.114			
CRP	1.040	1.011–1.070	0.006	1.038	1.001–1.076	0.049
LDH	1.003	0.999–1.008	0.124			
Presence of extra-pulmonary organ failure	8.500	1.964–36.790	0.004	13.126	1.608–107.161	0.016
APACHE II score	1.142	0.973–1.339	0.104			
SAPS II score	1.184	1.039–1.349	0.011	1.081	0.884–1.320	0.449
SOFA score	3.585	1.522–8.443	0.003			
High-dose steroids	2.597	0.769–8.775	0.124			

Age, sex, PaO_2_/ FiO_2,_ CRP, presence of extra-pulmonary organ failure and SAPS II score were included in the multivariate logistic regression analysis. AEIPF = acute exacerbation of idiopathic pulmonary fibrosis; PaO_2_ = arterial oxygen tension; FiO_2_ = fractional inspired oxygen; WBC = white blood cell; CRP = C-reactive protein; APACHE II = Acute physiology and chronic health evaluation II; SAPS II = simplified acute physiological score II; SOFA II = sequential organ failure assessment.

## Discussion

The study showed a majority (34/45, 75.6%) of AEIPF patients had stayed in RICU with a high hospital mortality rate (25/45, 55.6%). Occurrence of extra-pulmonary organ failure (18/45, 40.0%) was common in patients with AEIPF. More non-survivors (60.0%) developed multiple organ failures compared to survivors (15.0%). Elevated serum CRP level and presence of extra-pulmonary organ failures were associated risk factors for hospital mortality in AEIPF. These findings implicated an association between systemic inflammation and poor outcome in AEIPF.

The outcome of AEIPF was poor, and no effective treatment options were available^3,9–11^. A 10-year observational study showed that hospital mortality of AEIPF was 56.9% and 3-month mortality was 63.8%^11^. Saydain et al. reported that 61% of patients with AEIPF died during hospitalization and 92% of survivors died within 2 months after discharge^9^. The study from Song et al. also showed that hospital mortality in AEIPF was approximately 50.0%^3^, and in patients who were admitted to ICU, it exceeded 90%^12^. Various potential therapies had been reported in patients with AEIPF^13,14^. Unfortunately, these studies were mostly with small sample sizes and uncontrolled. Still, controversies remained over the use of corticosteroids and immunosuppressive agents in patients with AEIPF. A study from Japan demonstrated that early use of high-dose steroids could significantly increase survival rate^15^. But study from Papiris et al. showed corticosteroids therapy had no obvious advantage to 1-year survival^16^. International guidelines made a weak recommendation for the use of corticosteroids in AEIPF^1^. Results from clinical trials of nintedanib and pirfenidone suggested that IPF therapies may be helpful to prevent the development of AEIPF, but these data were from observational study^17–19^. A well-designed randomized clinical trial is still needed.

In the current study, CRP was identified as an independent risk factor of hospital mortality in AEIPF, which was consistent with previous studies^3^. Viral infection was reported to cause exacerbation of established pulmonary fibrosis in a murine model^20^. Lung microbiome, especially Staphylococcus and Streptococcus genera, showed an association with progression of IPF^21^. These studies suggested that infection or inflammation could be one pathogenic mechanism contributing to the occurrence of AE. More recently, a study from Li HP et al. also demonstrated that inflammatory cytokines, such as IL-6, IFN-γ, MIG, IL-17 and IL-9, were increased in the serum of AE-IPF compared with stable cases^22^, indicating inflammation rather than an accelerated intrinsic fibrotic process played a role in pathogenesis of AEIPF.

AEIPF shared several pathophysiological characteristics with ARDS^4^. Previous studies had noted that extra-pulmonary organ failures had increased the mortality risk of ARDS^23^. Sepsis syndrome/ multiple organ failures (MOFs) were the main cause of ARDS deaths^24^. Besides, higher APACHE II scores were reported to be associated with increased mortality in ARDS^25^. Similarly, this study showed that the presence of extra-pulmonary organ failure was a prognostic factor of AEIPF. It seemed that, during AEIPF, inflammation in lung triggered enhanced systemic inflammatory response leading to MOFs, and then worsened the clinical outcomes. The values of life supportive care and anti-inflammation therapy were also to be elucidated.

Notably, in our cohort, a majority of non-survivors (22/25, 88.0%) received NIV, and only 2 of them were performed IMV. Whether to withhold life sustaining therapies in patients with AEIPF is indeed a question. The available data suggested poor outcome for IPF patients who required NIV or IMV, although they temporarily corrected PaO_2_/FiO_2_ and reduced respiratory rate^26,27^. The hospital mortality of AEIPF who required MV was about 90%^28,29^. A systematic review comprising 9 single-center studies reported that 87.0% of IPF patients who had received IMV died in hospital^30^. NIV seemed more promising. Rush et al. revealed AEIPF patients who underwent NIV had a lower mortality rate (30.9%) compared to those who underwent IMV (51.6%)^31^. An early application of NIV when the general conditions of patients were less severe and the prevention of ventilator-associated pneumonia would benefit the survival^4^. However, ventilator-induced lung injury (VILI), which was an independent predictor for NIV failure^32^, should be acknowledged. In addition, excessive spontaneous effort and deflation injury resulted from NIV also caused lung injury^33^.

Several studies evaluated clinical predictors of mortality in patients with AEIPF, but a universally accepted predicted model did not exist. Akira et al. reported worse survival was associated with diffuse pattern on HRCT^34^. Lower forced vital capacity (FVC) and diffusion capacity for carbon monoxide (DLCO) before AE was observed in non-survivors compared to survivors^35^. Several prognostic biomarkers identified to be related with survival included increased Krebs Von den Lungen-6 (KL-6)^11^, higher hyaluronan^36^ and anti-heat shock protein 70 autoantibodies^37^. Recently, a staging system with four parameters, serum lactate dehydrogenase (LDH) level, serum KL-6, PaO_2_/FiO_2_, and total extent of abnormal findings on HRCT, was proposed to predict prognosis^11^. Prognostic model which considers pathologic physiology of various organs are acquired to provide guidance on early intervention.

This study had several limitations. First, the data was retrospectively collected from a single-center study. The selection bias was evitable and the small sample size weakened the power of the study. Second, the pulmonary function tests data were incomplete partly because of the critical illness so we could not assess the association between the last pulmonary function data before AE with the mortality.

## Conclusion

The outcome of AEIPF remained poor with a high in-hospital mortality and occurrence of extra-pulmonary organ failure was common. Elevated CRP and extra-pulmonary organ failure were independently associated with hospital mortality. Further investigations were still needed to clarify the association between systemic inflammation and AEIPF.

## Methods

### Patients

Patients diagnosed with AEIPF from July 2014 to September 2018 in Department of Pulmonary and Critical Care Medicine, Nanjing Drum Tower Hospital were enrolled. Electronic medical records were retrospectively reviewed. AEIPF was diagnosed according to the newly updated international guidelines^38^. The criteria were as follows: (1) previous or concurrent diagnosis of IPF with HRCT showing typical usual interstitial pneumonias (UIP) pattern; (2) acute worsening of dyspnea within one month; (3) HRCT showing new bilateral ground-glass opacity and/or consolidation superimposed on a background of UIP pattern and (4) exclusion of cardiac failure or fluid overload. Patients with incomplete data were excluded.

## Data collection

Clinical information was collected, including demographics, smoking history, clinical symptoms, duration from diagnosis of IPF to the occurrence of AE, treatment course and lengths of stay in RICU and hospital. The laboratory data included PaO_2_/ FiO_2_, blood routine, CRP, erythrocyte sedimentation rate (ESR), LDH and lymphocyte subset etc. APACHE II score^6^ and SAPS II^7^ in the first 24 h after admission were calculated. The presence of extra-pulmonary organ failures (coagulation, liver, cardiovascular, central nervous system and renal) were defined according to criteria of SOFA system^8^. Chest HRCTs of all patients in supine position were performed at admission. The newly appeared parenchymal abnormalities were classified into peripheral, multifocal and diffuse patterns based on the distributions^34^. Treatment information included usages and courses of corticosteroids, acetylcysteine, pirfenidone/nintedanib and respiratory support. All patients were administrated with oxygen therapy firstly. NIV was initiated when respiratory rate was more than 30 breaths/min or PaO_2_/FiO_2_ was less than 200mmHg^26^. The primary endpoint was hospital mortality. The length of stay in hospital was calculated from the date of admission to the date of discharge or death.

### Statistical analysis

Data was presented as mean with standard deviation for continuous variables or frequencies with percentages for categorical variables. T-test or the Mann–Whitney *U*-test was used for continuous variables. Chi-square test was used for categorical variables. Binary logistic regression analysis was used to evaluate the association between variables and mortality. Survival analysis was estimated using Kaplan–Meier curves and was compared between groups using the log-rank test. For continuous variables, the median was used as cut-off value. P value less than 0.05 was considered statistically significant. All statistical analysis was conducted through IBM SPSS Statistics version 23.0 (SPSS, Inc., Chicago, IL, USA) and Prism 8.0 (GraphPad, Inc., La Jolla, CA, USA).

### Ethical considerations

The patient data for this study were recorded by the authors. This study was approved by Ethics Committee of Nanjing Drum Tower Hospital, Clinical College of Nanjing Medical School, Jiangsu, China. Written informed consents were obtained from all subjects and the study was performed in accordance with the relevant guidelines/regulations.

## Data Availability

Data can be submitted by the corresponding author in case of a request.

## References

[1] Raghu G (2011). An official ATS/ERS/JRS/ALAT statement: idiopathic pulmonary fibrosis: evidence-based guidelines for diagnosis and management. Am. J. Respir. Crit. Care Med..

[2] Collard HR (2007). Acute exacerbations of idiopathic pulmonary fibrosis. Am. J. Respir. Crit. Care Med..

[3] Song JW, Hong SB, Lim CM, Koh Y, Kim DS (2011). Acute exacerbation of idiopathic pulmonary fibrosis: incidence, risk factors and outcome. Eur. Respir. J..

[4] Marchioni A (2018). Acute exacerbation of idiopathic pulmonary fibrosis: lessons learned from acute respiratory distress syndrome?. Crit Care.

[5] Papiris SA (2018). High levels of IL-6 and IL-8 characterize early-on idiopathic pulmonary fibrosis acute exacerbations. Cytokine.

[6] Knaus WA, Draper EA, Wagner DP, Zimmerman JE (1985). APACHE II: a severity of disease classification system. Crit. Care Med..

[7] Le Gall JR, Lemeshow S, Saulnier F (1993). A new Simplified Acute Physiology Score (SAPS II) based on a European/North American multicenter study. JAMA.

[8] Vincent JL (1996). The SOFA (Sepsis-related Organ Failure Assessment) score to describe organ dysfunction/failure. On behalf of the Working Group on Sepsis-Related Problems of the European Society of Intensive Care Medicine. Intensive Care Med..

[9] Saydain G (2002). Outcome of patients with idiopathic pulmonary fibrosis admitted to the intensive care unit. Am. J. Respir. Crit. Care Med..

[10] du Bois RM (2011). Ascertainment of individual risk of mortality for patients with idiopathic pulmonary fibrosis. Am. J. Respir. Crit. Care Med..

[11] Kishaba T, Tamaki H, Shimaoka Y, Fukuyama H, Yamashiro S (2014). Staging of acute exacerbation in patients with idiopathic pulmonary fibrosis. Lung.

[12] Rangappa P, Moran JL (2009). Outcomes of patients admitted to the intensive care unit with idiopathic pulmonary fibrosis. Crit. Care Resusc..

[13] Kataoka K (2015). Recombinant human thrombomodulin in acute exacerbation of idiopathic pulmonary fibrosis. Chest.

[14] Sakamoto S (2010). Cyclosporin A in the treatment of acute exacerbation of idiopathic pulmonary fibrosis. Intern. Med..

[15] Atsumi K (2018). Prognostic factors in the acute exacerbation of idiopathic pulmonary fibrosis: a retrospective single-center study. Intern. Med..

[16] Papiris SA (2015). Survival in Idiopathic pulmonary fibrosis acute exacerbations: the non-steroid approach. BMC Pulm. Med..

[17] Noble PW (2011). Pirfenidone in patients with idiopathic pulmonary fibrosis (CAPACITY): two randomised trials. Lancet.

[18] Taniguchi H (2010). Pirfenidone in idiopathic pulmonary fibrosis. Eur. Respir. J..

[19] Azuma A (2005). Double-blind, placebo-controlled trial of pirfenidone in patients with idiopathic pulmonary fibrosis. Am. J. Respir. Crit. Care Med..

[20] McMillan TR (2008). Exacerbation of established pulmonary fibrosis in a murine model by gammaherpesvirus. Am. J. Respir. Crit. Care Med..

[21] Han MK (2014). Lung microbiome and disease progression in idiopathic pulmonary fibrosis: an analysis of the COMET study. Lancet Respir. Med..

[22] Weng D (2019). The role of infection in acute exacerbation of idiopathic pulmonary fibrosis. Mediators Inflamm.

[23] Luo L (2017). Clinical predictors of hospital mortality differ between direct and indirect ARDS. Chest.

[24] Ferring M, Vincent JL (1997). Is outcome from ARDS related to the severity of respiratory failure?. Eur. Respir. J..

[25] Stapleton RD (2005). Causes and timing of death in patients with ARDS. Chest.

[26] Vianello A (2014). Noninvasive ventilation in the event of acute respiratory failure in patients with idiopathic pulmonary fibrosis. J. Crit. Care.

[27] Mollica C (2010). Mechanical ventilation in patients with end-stage idiopathic pulmonary fibrosis. Respiration.

[28] Blivet S (2001). Outcome of patients with idiopathic pulmonary fibrosis admitted to the ICU for respiratory failure. Chest.

[29] Fumeaux T, Rothmeier C, Jolliet P (2001). Outcome of mechanical ventilation for acute respiratory failure in patients with pulmonary fibrosis. Intensive Care Med.

[30] S. Mallick, Outcome of patients with idiopathic pulmonary fibrosis (IPF) ventilated in intensive care unit. *Respiratory Med. 102*, 1355–1359, doi:10.1016/j.rmed.2008.06.003 (2008) 10.1016/j.rmed.2008.06.003 18635345

[31] Rush B, Wiskar K, Berger L, Griesdale D (2016). The use of mechanical ventilation in patients with idiopathic pulmonary fibrosis in the United States: a nationwide retrospective cohort analysis. Respir Med.

[32] Carteaux G (2016). Failure of noninvasive ventilation for de novo acute hypoxemic respiratory failure: role of tidal volume. Crit Care Med.

[33] Katira BH (2019). Ventilator-induced lung injury: classic and novel concepts. Respir. Care.

[34] Akira M, Kozuka T, Yamamoto S, Sakatani M (2008). Computed tomography findings in acute exacerbation of idiopathic pulmonary fibrosis. Am. J. Respir. Crit. Care Med..

[35] Simon-Blancal V (2012). Acute exacerbation of idiopathic pulmonary fibrosis: outcome and prognostic factors. Respiration.

[36] Inokoshi Y (2013). Clinical significance of serum hyaluronan in chronic fibrotic interstitial pneumonia. Respirology.

[37] Kahloon RA (2013). Patients with idiopathic pulmonary fibrosis with antibodies to heat shock protein 70 have poor prognoses. Am. J. Respir. Crit. Care Med.s.

[38] Collard, H. R. et al. Acute exacerbation of idiopathic pulmonary fibrosis. An international working group report. *Am J Respir Crit Care Med* **194**, 265–275, doi:10.1164/rccm.201604–0801CI (2016). 10.1164/rccm.201604-0801CI 27299520

